# Optical Assay of the Functional Impact of Cuprizone-Induced Demyelination and Remyelination on Interhemispheric Neural Communication in the Anterior Cingulate Cortex via the Corpus Callosum

**DOI:** 10.1523/ENEURO.0511-24.2024

**Published:** 2025-01-03

**Authors:** Kyoka Tsukuda, Yoko Tominaga, Makiko Taketoshi, Michiko Miwa, Kentaro Nakashima, Takashi Tominaga

**Affiliations:** ^1^Graduate School of Pharmaceutical Science, Tokushima Bunri University, Sanuki 769-2193, Japan; ^2^Institute of Neuroscience, Tokushima Bunri University, Sanuki 769-2193, Japan; ^3^Kagawa School of Pharmaceutical Sciences, Tokushima Bunri University, Sanuki 769-2193, Japan

**Keywords:** anterior cingulate cortex, corpus callosum, cuprizone, medial prefrontal cortex, multiple sclerosis, voltage-sensitive dye

## Abstract

Cuprizone (CPZ) is a widely used toxin that induces demyelinating diseases in animal models, producing multiple sclerosis (MS)-like pathology in rodents. CPZ is one of the few toxins that triggers demyelination and subsequent remyelination following the cessation of its application. This study examines the functional consequences of CPZ-induced demyelination and the subsequent recovery of neural communication within the anterior cingulate cortex (ACC), with a particular focus on interhemispheric connectivity via the corpus callosum (CC). By employing wide-field, high-speed, voltage-sensitive dye imaging, we were able to provide real-time mapping of neural activity in the ACC of CPZ-fed mice. Although we could not record physiological signals from the CC, the results demonstrated a notable impairment in interhemispheric connections within the ACC via the CC, with the most pronounced loss observed in a specific coronal slice among a series of slices examined. Notably, the latency of neural signal propagation remained largely unaltered despite connectivity loss, indicating that demyelination affects the extent, rather than the temporal dynamics, of neural communication. It is noteworthy that while functional connectivity appeared to recover fully after the cessation of CPZ, histological analysis revealed only partial recovery of myelination, indicating a discrepancy between functional and structural recovery. These findings enhance our understanding of how demyelination affects the ACC's role in orchestrating neural activity, particularly in light of the slice-specific nature of interhemispheric communication impairments. These findings offer new insights into MS pathology, particularly regarding the role of the CC in interhemispheric communication and potential therapeutic strategies.

## Significance Statement

Cuprizone (CPZ) is widely used to model multiple sclerosis (MS) in rodents by inducing demyelination. While the demyelination effects of CPZ have been widely studied, this study explores CPZ’s impact on the prefrontal cortex (PFC). Using voltage-sensitive dye imaging (VSDI), we identified disruptions in PFC connectivity within and between hemispheres in CPZ-fed mice, though signal timing remained unaffected. This finding suggests that demyelination impairs connectivity without slowing transmission speed. Remarkably, connectivity restoration aligned with brain remyelination, providing insights into recovery pathways in MS. This study not only demonstrates VSDI’s potential to detect functional impairments but also uncovers CPZ’s broader effects on brain connectivity, highlighting new therapeutic opportunities.

## Introduction

Cuprizone (CPZ; bis-cyclohexanone-oxaldihydrazone) is widely used for inducing demyelination in rodent models and is particularly valuable for studying demyelination-associated diseases, such as multiple sclerosis (MS; Lucchinetti et al., 2000, 1999). CPZ’s demyelinating effects are prominent across various brain regions, with particular vulnerability observed in the corpus callosum (CC; Hiremath et al., 1998; Jurevics et al., 2002, 2001; Vega-Riquer et al., 2019), a major structure responsible for communication between both cerebral hemispheres, and this targeted demyelination significantly impairs higher brain functions. Unlike other MS models, CPZ administration not only induces demyelination but also allows for remyelination upon treatment cessation, offering a unique platform to study both processes (Matsushima and Morell, 2001; Praet et al., 2014). In contrast, other toxin-induced models, such as experimental autoimmune encephalomyelitis, focus mainly on inflammatory mechanisms and do not provide opportunities to study remyelination (Storch et al., 1998).

The CC is vital for interhemispheric communication, integrating information between the brain’s left and right hemispheres (Aboitiz and Montiel, 2003; Rovira and Geijo-Barrientos, 2016). CPZ-induced damage to the CC disrupts this interhemispheric connectivity, leading to impairments in cognitive and motor functions, including motor coordination (Li et al., 2016) and cognitive processing (Gazzaniga, 2005; Tovar-Moll et al., 2007; Liu et al., 2010; Fenlon and Richards, 2015; Huang et al., 2015; Höller-Wallscheid et al., 2017; Degraeve et al., 2022; Russo et al., 2022; Fabri and Polonara, 2023).

The anterior cingulate cortex (ACC), an essential part of the prefrontal cortex (PFC; Fuster, 2001; Merre et al., 2021), relies heavily on the CC to coordinate activity between the hemispheres. The ACC is involved in a range of functions, including cognitive processes, emotional regulation, and integration of affective information, all of which depend on intact CC connectivity for effective interhemispheric coordination (Rolls et al., 2022). Consequently, damage to the CC directly impairs the ACC's ability to synchronize activity across the hemispheres, potentially leading to disruptions in both cognitive and emotional processing.

A previous work successfully visualized neuronal activity propagation within the ACC, encompassing both intra- and interhemispheric connections, using single-photon wide-field voltage–sensitive dye imaging (VSDI; Gusain et al., 2023). The VSDI, first developed in the 1970s (Salzberg et al., 1973; Cohen et al., 1978; Loew et al., 1992; Knöpfel and Song, 2019; Milicevic et al., 2023), has been refined to serve as a quantitative tool for wide-field recording of neuronal activity (Suh et al., 2011; Gusain et al., 2023; Tominaga et al., 2023; Utsumi et al., 2023; Ojima et al., 2024). Although VSDI cannot record physiological data from the CC because of technical limitations, the ACC, which plays a central role in coordination of cognitive and affective responses, remains an ideal target for studying CPZ-induced demyelination using VSDI to capture its functional dynamics.

In this study, we aim to assess how CPZ-induced demyelination affects both intra- and interhemispheric neural communication within the ACC. Although substantial research has been conducted on the structural effects of CC demyelination, functional assessments of the interhemispheric connectivity are relatively rare. Few studies have specifically examined the real-time effects of demyelination on neural communication between hemispheres, particularly in the context of the ACC (Bando et al., 2008; Crawford et al., 2009). By applying our VSDI technique, we aim to provide a detailed, quantitative analysis of neuronal activity within the ACC and its propagation across the CC during both demyelination and remyelination phases. Understanding these functional disruptions is essential for advancing knowledge of central nervous system (CNS) demyelination and identifying potential therapeutic targets.

## Materials and Methods

### Animals

C57BL/6J male mice, aged 8 weeks, were obtained from a local distributor (SLC). They were housed in the animal facilities under a 12 h light/dark cycle with controlled temperature (20–24°C) and humidity (50–60%). All animal experiments were approved by the Animal Care and Use Committee of Tokushima Bunri University (approval numbers, KP23-83–2 and KP24-83–2), adhering strictly to all applicable international, national, and institutional guidelines for the care and use of animals.

### CPZ diet administration

CPZ (catalog #04198-30, Kanto Chemical) was added to the powdered feed (Powder CE-2, CLEA Japan) to create a 0.3% (*w*/*w*) CPZ-containing feed (Lindner et al., 2008). Mice were divided into three groups: a CPZ group, a control group, and a recovery group. Mice in the CPZ group were fed 0.3% CPZ-containing powdered feed for 6 weeks with *ad libitum* access to food and water (hereafter referred to as CPZ mice). Age-matched mice in the control group were fed powdered feed without CPZ for the same period (hereafter referred to as control mice). To assess the recovery effects after CPZ treatment, we switched back the diet for the recovery group to the standard solid feed for an additional 5 weeks following the 6 week CPZ diet (Matsushima and Morell, 2001; Lindner et al., 2008; hereafter referred to as REC mice).

### Preparation of brain slices and VSD staining

**Figure 1.**
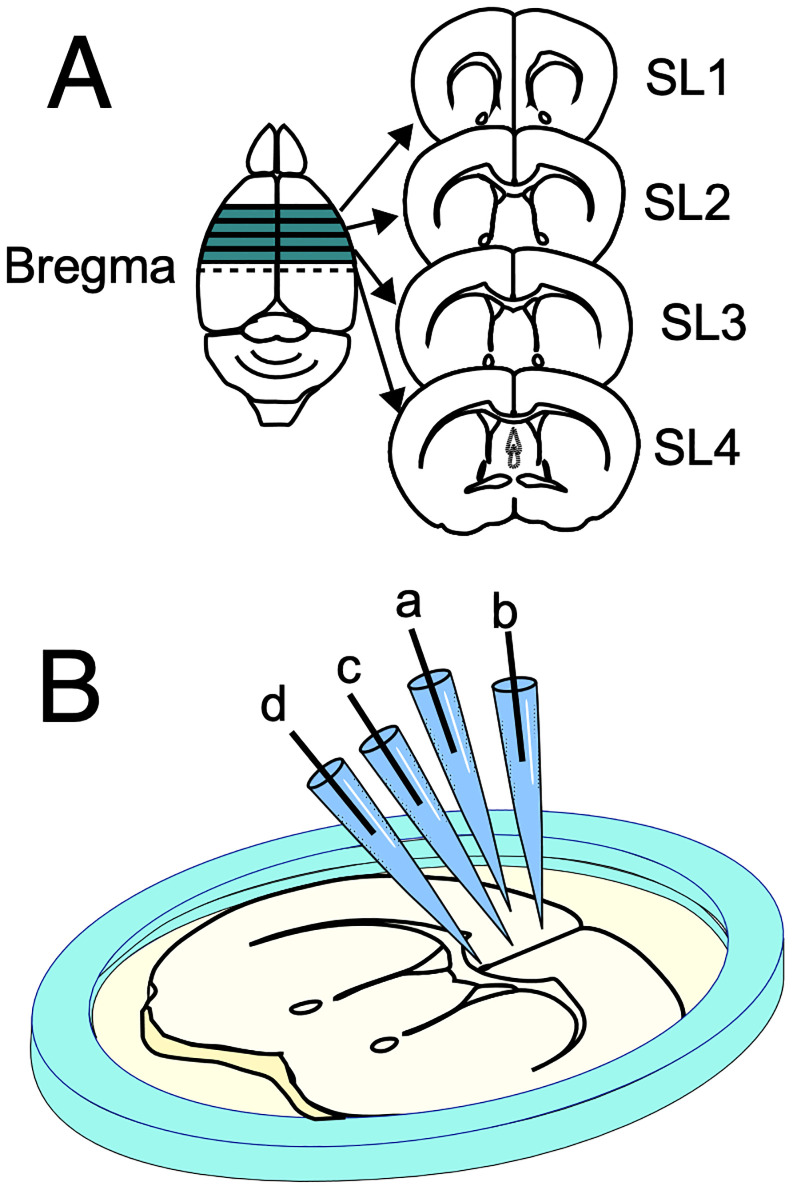
***A***, Consecutive coronal slices from the rostral to caudal part of the PFC. These slices represent different regions of the PFC, moving from the rostral (front) to the caudal (back) regions of the brain. ***B***, Schematic illustration of an SL3 slice placed on a ring with a membrane filter. The diagram shows four stimulation sites (a–d) along Layers II/III of the cortex, each representing different regions targeted for optical recording during experiments.

All experimental procedures for optical recording with VSD were conducted in accordance with recent publications (Tominaga et al., 2019; Gusain et al., 2023). Briefly, mice were anesthetized with isoflurane, after which the brain was immediately resected and placed in a cold artificial cerebrospinal fluid (ACSF) solution (in mM: 124 NaCl, 2.5 KCl, 2 CaCl_2_, 2 MgSO_4_, 1.25 NaHPO_4_, 26 NaHCO_3_, and 10 glucose), pH 7.4, for 5 min. Subsequently, a block containing the PFC was sectioned into 350-µm-thick coronal slices using a vibrating slicer (VT-1000 or VT-1200; Leica Microsystems; Fig. 1*A*). Each slice was transferred to a specialized holder with a membrane filter (Tominaga et al., 2000, 2019) designed to keep the slices viable and maintain their orientations. The slices were then compared with images from an atlas (Paxinos and Franklin, 2008) and labeled SL1–SL4 according to their distance from the bregma: 1.34, 0.98, 0.62, and 0.26 mm (Fig. 1*A*).

Four coronal slices (SL1–SL4) were collected from each animal. Slices were transferred to a humidified chamber with ACSF, continuously supplied with a 95% O_2_/5% CO_2_ gas mixture. After a 40 min incubation at 28°C, each slice was stained for 20 min with 110 µl of VSD solution and subsequently washed with ACSF. The VSD solution contained 0.2 mM Di-4-ANEPPS, 2.5% ethanol, 0.13% Cremophor EL, 1.17% distilled water, 48.1% fetal bovine serum, and 48.1% ACSF.

### Optical recording

Each slice was placed in a recording chamber with continuous perfusion of oxygenated ACSF, bubbled with a 95%/5% O_2_/CO_2_ gas mixture, at a 1 ml/min rate. To mimic in vivo cortical activity, we collected all data during perfusion with 1 µM SR95531 (gabazine; Tocris Bioscience); this method has been shown to effectively suppress excessive inhibitory synaptic transmission in slice conditions, thus preserving cortical activity (Iijima et al., 1996; Kajiwara et al., 2019).

Epifluorescence optics with two lenses were used to visualize the slices: a 5× objective lens for a stereo microscope MZ series (#10447242, Leica Microsystems) and a tube lens (F1.4 85 mm, SAMYANG). Stained slices were illuminated with an excitation light from a stabilized LED source (LEX2-LZ4; Brainvision) filtered at 530 ± 30 nm. Fluorescence from the stained sections was passed through an emission filter (>590 nm) and projected onto a camera (MiCAM03, Brainvision). This setup allowed for high-frame–rate recordings of neural activity across the entire coronal slice (1 ms/frame, unless otherwise stated) at a high spatial resolution (256 × 256 pixels).

A microcapillary glass electrode (outer diameter, 1.0 mm; inner diameter, 0.75 mm) filled with ACSF solution was used for stimulation, along with a ground electrode filled with 3 M KCl solution connected to an AgCl-coated Ag wire.

The data were analyzed using the BV_Ana software (Brainvision) and a custom lab-made macroprogram in Igor Pro (ver. 9, WaveMetrics). A specially designed macro within Igor Pro was used for numerical and statistical analyses, and one-way ANOVA (fixed-effect model) was applied for all statistical evaluations. Statistical significance was set at *p* < 0.05.

Electrical stimulation (bipolar, 40 V, 0.5 ms each) was applied at four different sites (Fig. 1*B*, a–d) using a stimulator (ESTM-8; Brainvision). Throughout the experiment, the cortex subjected to electrical stimulation was referred to as the ipsilateral cortex, while the opposite side was referred to as the contralateral cortex.

### Reslicing for histochemical and immunohistochemical analysis

Slices used for VSD recording were fixed in 4% paraformaldehyde in 0.1 M phosphate buffer, pH 7.2 (4°C). The slices were then rinsed with 0.1 M phosphate-buffered saline (PBS), pH 7.2, and cryoprotected with 30% sucrose in PBS. Each slice was placed on a flat surface of iced embedding material (Tissue-Tek O.C.T. Compound, 4583, Sakura Finetek Japan) within a cryostat (HM525NX, Thermo Fisher Scientific). Slices were then resectioned into thin (12 µm) sections in 0.01 M PBS, pH 7.2, for subsequent histological and immunohistochemical analysis.

### Myelin staining of the CC with FluoroMyelin green

FluoroMyelin Green (FMG; F34651, Thermo Fisher Scientific) diluted 300× in PBS (0.2%Triton–PBS) containing 0.2 Triton X-100 (Thermo Fisher Scientific, lot. number j821818) was used to stain the thin slices overnight at 4°C.

### Immunofluorescence staining of the cortical tissue with anti-PLP (proteolipid protein) antibody

Slices were immersed in normal donkey serum (Jackson ImmunoResearch Laboratories) diluted to 5% in PBS containing 0.2% Triton X-100 (0.2% Triton–PBS) for 1 h. After washing with PBS, the slices were incubated with rabbit anti-myelin PLP primary antibody. An anti-PLP antibody against a synthetic peptide (CGRGTKF) corresponding to the C-terminus of human and mouse PLP was produced in our laboratory. This peptide was coupled to keyhole limpet hemocyanin at the N-terminal cysteine residue for rabbit immunization.

The slices were then incubated at room temperature for 2 h with an Alexa Fluor 488-conjugated donkey anti-rabbit IgG secondary antibody (Jackson ImmunoResearch Laboratories; 711-546-152) diluted 1:200 in 0.2% Triton–PBS, along with a nuclear detection probe (TO-PRO-3 iodide, Thermo Fisher Scientific, T3605) diluted 1:500.

FMG and immunofluorescence-stained slices were mounted in nonfluorescent glycerol (66.7% glycerol, 0.167 M sodium carbonate buffer), pH 8.6, and analyzed with a confocal laser scanning microscope (FV1000, Olympus). Images were processed using Fiji (version 2.14.0/1.54f), a distribution of the ImageJ software (Schindelin et al., 2012).

## Results

VSD preferentially incorporates into lipid-rich components, leading to elevated background fluorescence in myelinated axons, such as those in the CC. Consequently, we did not record physiological data from the CC because of elevated background fluorescence. Instead, we focused on intra- and interhemispheric neural signal propagation within the ACC.

### CPZ-induced demyelination inhibits the interhemispheric spread of neuronal activity

**Figure 2.**
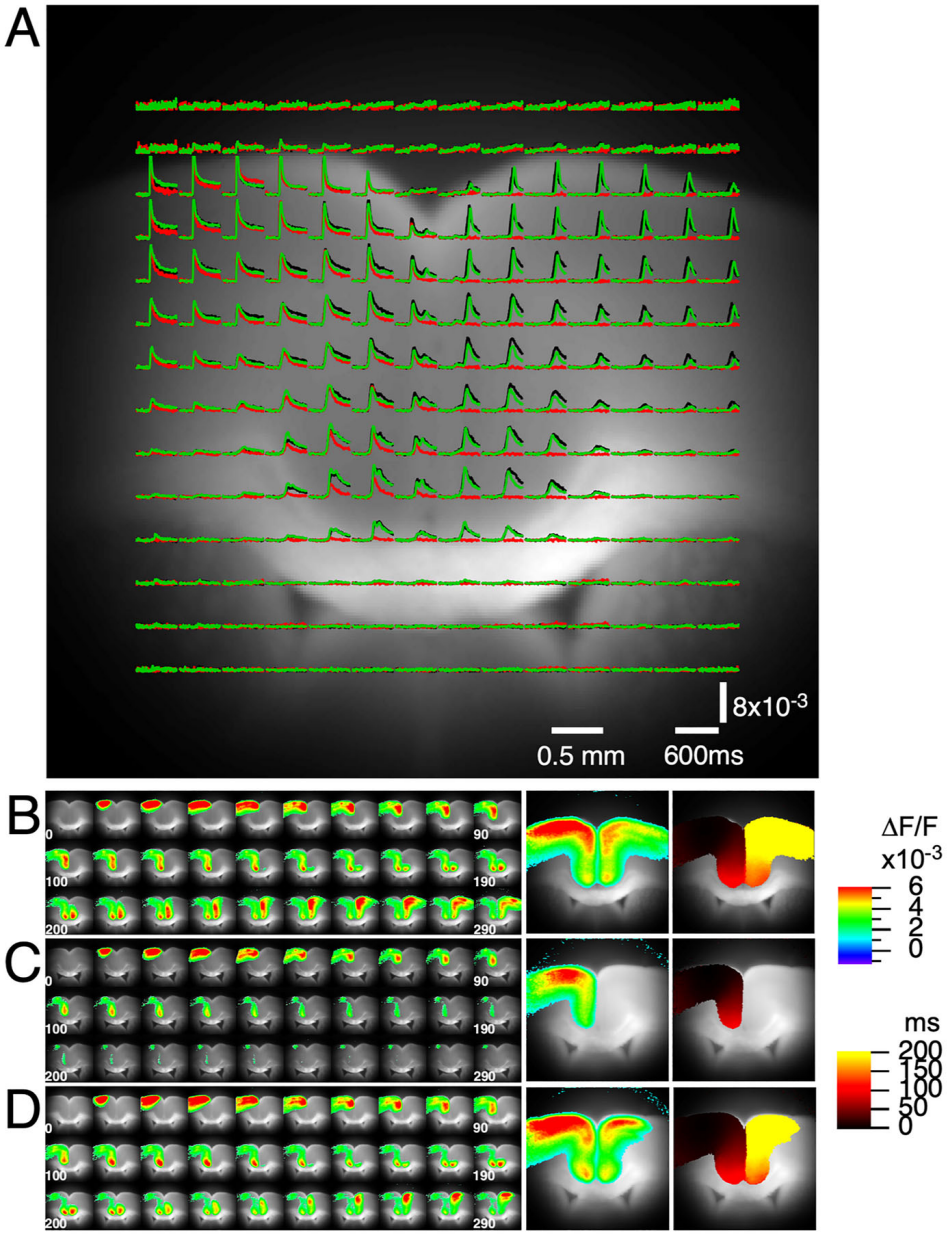
Representative responses to electrical stimulation in Layers II/III of the ACC of a coronal slice. ***A***, A fluorescent image of the ACC coronal slice obtained using a high-speed 256 × 256 imaging system. Superimposed on this image are traces representing optical signals measured from selected pixels. Black traces correspond to data from control mice, red traces represent CPZ mice, and green traces represent REC mice. ***B***, Consecutive images illustrate the propagation of neuronal activity following electrical stimulation, shown in increments of 10 ms. Adjacent to these images is a panel showing the projection of the peak signal values for each pixel onto the original fluorescent image, highlighting the amplitude of the response. The rightmost panel displays the latency values from the time of stimulation, demonstrating the rate at which activity spread across the slice. ***C, D***, Similar datasets are presented for CPZ mice (panel ***C***) and REC mice (panel ***D***), highlighting the differences in activity spread and propagation time compared with control mice.

Electrical stimulation of Layers II/III of the ACC on the dorsal (the upper part, nearer to the crown of the skull) side of a coronal slice (Fig. 1*B*, stimulation “a” on the SL3 slice) induced neuronal activity propagation along the ACC, progressing from the dorsal side toward the medial (closer to the midline) and ventral (the lower part, nearer to the base of the skull) side. The activity propagated laterally toward the motor cortex at the dorsal cortex. The excitation reached the end of the ACC near the CC and subsequently spread to the opposite hemisphere, appearing at the most ventral side of the contralateral ACC. Representative pixel-wise optical signal traces are shown in Figure 2*A* (black traces), overlaid on a fluorescence image of the slice. Sequential images of activity spread are shown in Figure 2*B*. The mapped data represent projections of peak signal values at each pixel (amplitude map) and the time to reach 20% of these peak values (latency map) onto the fluorescence image, illustrating both the amplitude and latency of the activity (Fig. 2*B*, right panel).

The equivalent stimulation of the same coronal slice level in CPZ mice is shown in Figure 2*A* as a red trace. The corresponding consecutive images, along with amplitude and rise-time mapping, are shown in Figure 2*C*. Notably, the interhemispheric spread of activity was completely absent in the CPZ-treated slices. In REC mice, the interhemispheric spread was restored, as indicated by the green traces in Figure 2*A* and the images of activity spread in Figure 2*D*.

The ipsilateral side (left side in the figure where the stimulation was applied) in CPZ mice exhibited a smaller and shorter response than did that in the control and REC mice. This is evident when comparing the red trace to the black (control) and green (REC) traces in Figure 2*A*, as well as in panel C (CPZ) relative to panels B (control) and D (REC).

### The effect of the CPZ diet was most severe in SL3 while sparing other slices

**Figure 3.**
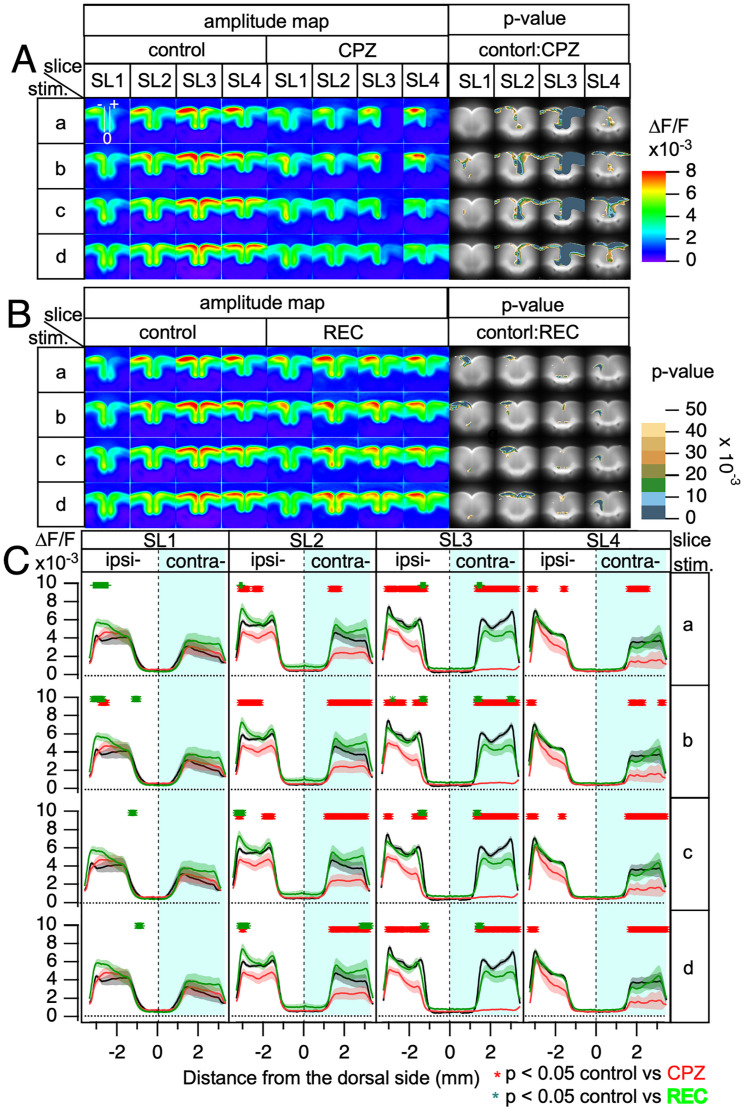
Averaged amplitude maps and line profiles for electrical stimulation across slices SL1 to SL4. ***A***, Averaged amplitude maps for stimulations a–d applied to slices SL1–SL4 are shown in pseudocolor. Each pixel value represents the response amplitude, and pixel-wise statistical analysis was performed using ANOVA to compare control and CPZ mice. The *p* values are represented in pseudocolor to highlight significant differences. ***B***, Averaged amplitude maps comparing the control and REC mice using the same pseudocolor representation and pixel-wise ANOVA analysis as shown in panel ***A***. ***C***, Line profiles of amplitude along a selected line are superimposed on the SL1-stim image, with data plotted for control (black line), CPZ (red line), and REC (green line) mice, along with the standard error of the mean (SEM). Statistically significant differences (*p* < 0.05) between the control and CPZ mice are indicated with red symbols, while the differences between the control and REC mice are denoted by green symbols positioned above the corresponding line profile graphs. In all the panels, stimulation was applied to the left hemisphere of the slices (ipsilateral side). *n* = 6–10 for each group.

Data were collected from five animals per group (control, CPZ, and REC mice), with four slices per animal, resulting in five slices for each slice level (SL1–SL4). Both sides of each slice were equally treated, and up to 10 averaged data points per slice level were obtained for evaluation of the effects of CPZ treatment. Figure 3A presents the averaged amplitude maps for slices SL1–SL4 in both the control and CPZ mice upon stimulation at Sites a to d. In these maps, stimulation was consistently applied on the left side of the figure (representing the ipsilateral cortex).

In the control slices, all amplitude maps demonstrated that the neuronal activity propagated from the stimulation site to both the ipsilateral (the same side of the brain as the stimulation) and contralateral sides (the opposite side). The interhemispheric spread was most pronounced in SL3 when stimulated at Sites b to d. Under these conditions, activity maps of the ipsilateral and contralateral sides were nearly symmetrical, indicating similar levels of neuronal activity on both sides. Specifically, the spread in the contralateral cortex was almost identical to that in the ipsilateral cortex. This symmetry was also evident in the peak amplitude profiles along the dorsal–ventral axis of the ACC, as indicated by the black lines in Figure 3*C*, which demonstrate nearly symmetrical propagation in SL3.

In the other slices (SL1, SL2, and SL4), the activity observed on the contralateral side was weaker than that on the ipsilateral side. This reduced activity was particularly evident in the dorsal (top) region of the slices. As illustrated in Figure 3*C*, this region appears on the far side from the center (0), emphasizing the diminished responses on the contralateral side. This observation may reflect the reduced interhemispheric connectivity in these slices (Gusain et al., 2023). When stimulation was applied to the ventral part of the ipsilateral cortex, it tended to induce more robust interhemispheric propagation to the contralateral side than did stimulation applied to the dorsal part. Despite the strong activity transfer, when this propagated activity reached the contralateral cortex, it did not spread effectively to the dorsal (top) region of that cortex.

In SL3, CPZ mice exhibited an almost complete blockade of interhemispheric propagation of activity. The ipsilateral side displayed minimal changes compared with the control mice, whereas the contralateral side exhibited virtually no response.

The *p* values from these pixel-wise statistical evaluations are depicted in pseudocolors in the rightmost panel of Figure 3*A* to illustrate the areas of significance. A statistically significant difference (*p* < 0.05; *n* = 6–10) was observed in the contralateral side of SL3 between control and CPZ mice. A significant decrease was observed at the most ventral edge of cg2 in the ipsilateral ACC in SL3. This change occurred alongside an almost complete loss of interhemispheric propagation, suggesting a notable alteration in local connectivity. Differences on the ipsilateral side were also evident in SL2 and SL3 within Layers II/III of the ACC.

Mice that underwent 5 weeks of recovery after CPZ cessation (REC mice) were also compared with control mice (Fig. 3*B*). In SL3, the impaired intrahemispheric propagation caused by CPZ was nearly fully recovered, with only a few statistical differences remaining compared with control mice. Overall, 5 weeks of returning to a regular diet resulted in nearly complete recovery from CPZ-induced impairments in both inter- and intrahemispheric connections.

In the line profiles of the amplitude maps (Fig. 3*C*), REC mice exhibited enhanced recovery, particularly in SL1 and SL2, where the amplitude on the dorsal ipsilateral side was significantly higher than that in the controls. This exaggerated response suggests possible cortical overcompensation, especially in dorsal regions.

### The effect of the demyelination on signal propagation delays

**Figure 4.**
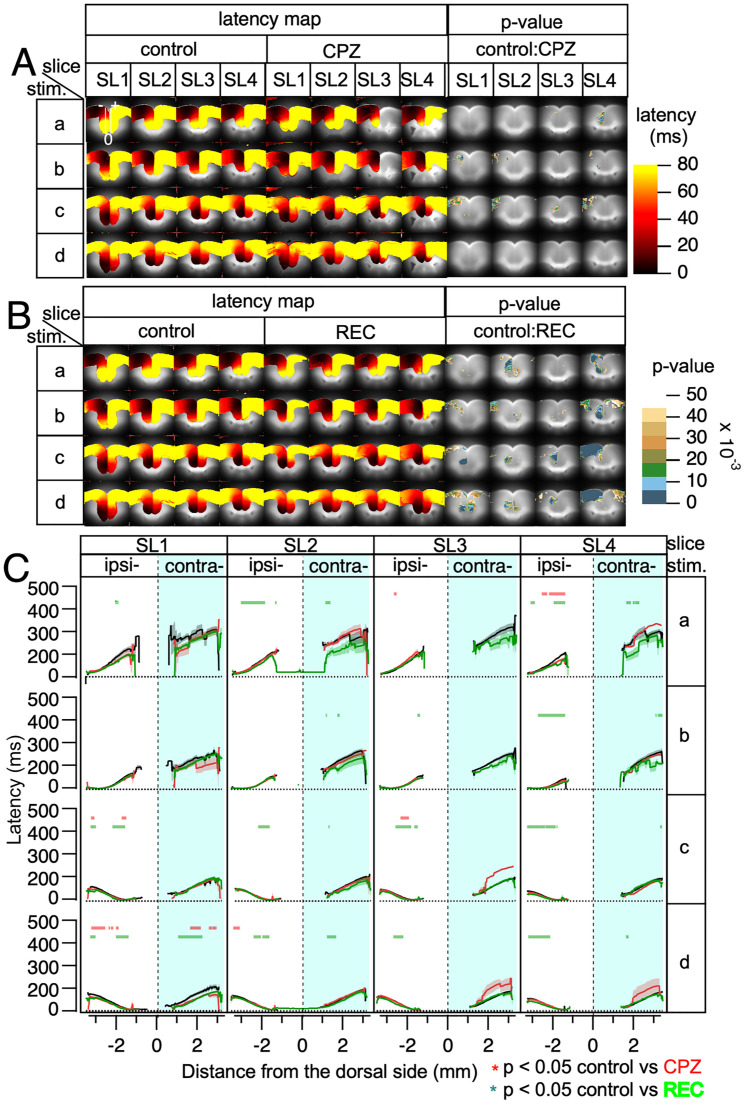
Averaged latency maps and line profiles for electrical stimulation across slices SL1–SL4. ***A***, Averaged latency maps following stimulations a–d in slices SL1–SL4 shown in pseudocolor. Each pixel value represents the response latency, and pixel-wise statistical comparisons were performed using ANOVA to compare the control and CPZ mice. Areas with significant latency differences are indicated by corresponding *p* values in pseudocolor. ***B***, Averaged latency maps comparing the control and REC mice using the same pseudocolor representation and pixel-wise ANOVA analysis as shown in panel ***A***. ***C***, Line profiles of latency along a selected line are superimposed on the SL1-stim image, with data plotted for the control (black line), CPZ (red line), and REC (green line) mice, including the SEM. Statistically significant differences (*p* < 0.05) between the control and CPZ mice are indicated with red symbols, while significant differences between the control and REC mice are marked with green symbols above the corresponding line profile graphs. In all the panels, electrical stimulation was applied to the left hemisphere of the slices (ipsilateral side). *n* = 6–10 for each group.

Figure 4 presents the averaged latency maps for control, CPZ, and REC mice, displayed in pseudocolor to represent time from the point of stimulation, in a pixel-wise manner. These maps illustrate latency variations across the cortex under different experimental conditions. In general, Figure 4 indicates that the latency patterns and the associated *p* values reveal no significant differences across most regions of the slices.

Even in SL3, where interhemispheric connections were almost entirely lost, the latency in the ipsilateral cortex did not exhibit significant differences compared with the control mice.

Plots along the indicated lines for both ipsilateral and contralateral cortices further confirmed these findings, showing consistent latencies with no significant deviations between groups.

### Demyelination and remyelination in the CC: histological analysis

**Figure 5.**
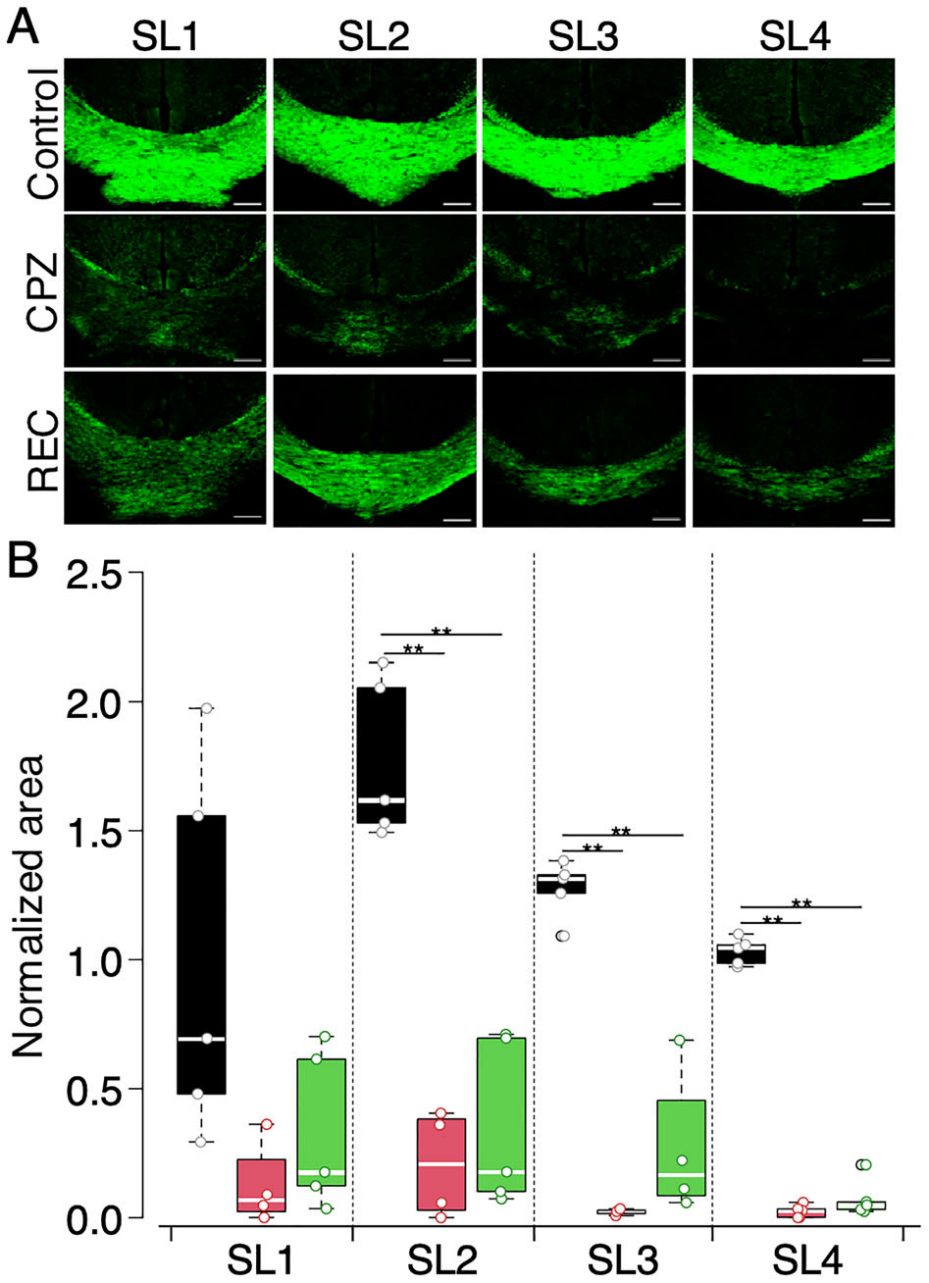
Myelin staining of slices used in VSDI experiments using FMG. ***A***, FMG fluorescent images of the CC areas in slices SL1–SL4 for control, CPZ, and REC mice. The images depict myelin content in each group, with reduced fluorescence indicating demyelination. Scale bar, 200 µm. ***B***, Box plots representing the normalized myelin area stained with FMG across the control, CPZ, and REC mice. Data are presented as black (control), red (CPZ), and green (REC). Significant differences are indicated (*p* < 0.01). *n* = 4–6 for each group.

Histological examinations were performed on the same slices used for VSDI after resectioning to a thickness of 12 µm. Figure 5*A* displays representative images of the CC stained with FMG for control, CPZ, and REC mice.

The CPZ mice exhibited significantly lower FMG fluorescence in the CC than did the control mice, indicating a marked decrease in myelination.

Figure 5*B* presents the normalized fluorescence area of FMG-stained CC for control, CPZ, and REC mice. The CPZ mice showed significantly decreased staining in SL2, SL3, and SL4, consistent with demyelination. In REC mice, after 5 weeks of a normal diet, the fluorescence area remained reduced compared with that in the control, indicating that the remyelination process was incomplete. This finding suggests that although some recovery occurred, it was insufficient to fully restore myelination to control levels, as evidenced by decreased FMG staining in the CC.

### Demyelination and remyelination in cortical regions: immunohistochemical analysis using anti-PLP antibody

**Figure 6.**
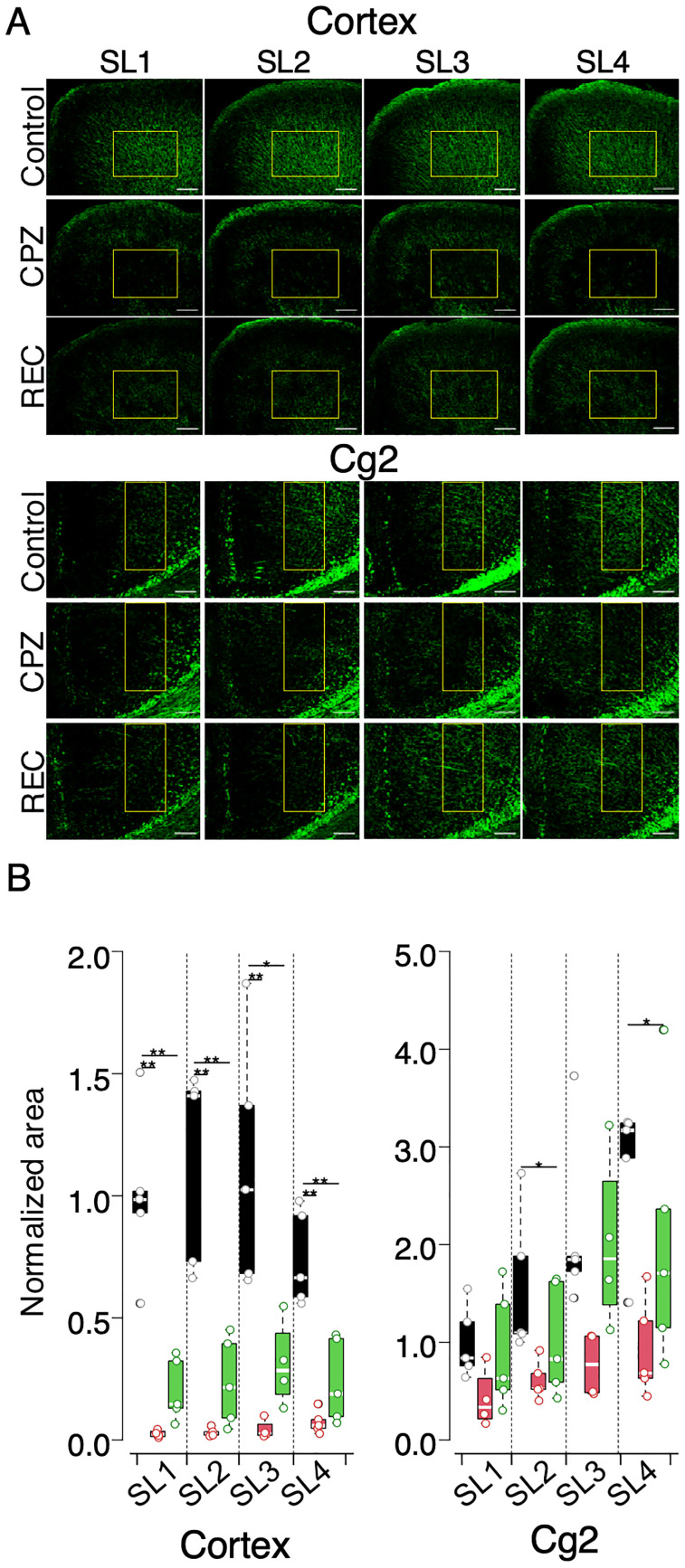
Myelin staining of slices used in VSDI experiments using PLP antibody. ***A***, PLP antibody fluorescent staining of slices used for VSDI experiments, targeting the dorsal motor cortex and ventral cortex (cg2 of the ACC) across slices SL1–SL4 in control, CPZ, and REC mice. Stained areas indicate the presence of myelin, with reduced staining suggesting demyelination. Scale bar, 200 µm (cortex), 100 µm (cg2). ***B***, The box plot representing the normalized area of fluorescence in the regions of interest (ROI, indicated by square boxes in panel ***A***). Data for control (black), CPZ (red), and REC (green) mice are shown, with significance levels indicated by *p < 0.05; **p < 0.01. *n* = 4–6 for each group.

To further investigate the extent of myelination in the cortical tissues, anti-PLP antibody staining was performed (Fig. 6). This analysis was conducted to more precisely quantify myelination in cortical areas, specifically targeting PLP, a key component of the myelin sheath (Lindner et al., 2008).

Figure 6*B* presents the results of anti-PLP antibody staining across SL1–SL4 slices. In CPZ mice, PLP levels were significantly lower than those in controls, particularly in the cortical areas adjacent to cg1. In contrast, the reduction in PLP expression in cg2 was less pronounced than that in cg1, though it remained statistically significant in both SL2 and SL4.

In REC mice, partial restoration of PLP levels was observed in the cortex. However, PLP expression remained significantly different from that in the control mice (Fig. 6*B*), indicating incomplete remyelination.

Notably, the structural recovery indicated by PLP staining did not fully correlate with the functional recovery observed in the VSD optical signals. While VSDI revealed substantial functional recovery in terms of signal propagation, the underlying myelin structure did not return to control levels, suggesting that the restoration of function may involve compensatory mechanisms independent of full remyelination, as discussed below.

## Discussion

This study demonstrates that CPZ impairs interhemispheric neural communication in the ACC, particularly at specific levels of coronal slices. Specifically, SL3 exhibited the most severe impairment in single-photon wide–field VSDI. Notably, despite the substantial restoration of interhemispheric activity propagation to levels comparable with those of the control after CPZ cessation, histological analysis revealed only partial remyelination, particularly in the CC. This discrepancy indicates that functional recovery does not strictly depend on complete structural recovery and highlights the need for physiological assays to understand neural resilience more comprehensively.

### Rostrocaudal position-dependent vulnerability and nonuniform effects

Among the coronal slices examined, impairment was most pronounced in SL3 (0.62 mm from the bregma), suggesting a position-dependent vulnerability to CPZ-induced demyelination in the ACC. This observed vulnerability does not necessarily indicate molecular or physiological uniqueness in this brain region. SL3 exhibited significantly stronger contralateral signal propagation than did SL2 and SL4, particularly in the dorsal region of the contralateral hemisphere in control slices. The comparable signal amplitudes on the ipsilateral side suggested minimal differences in the signal-to-noise ratio among the slices in VSDI. Instead, the intrinsic physiological mechanisms underlying this pronounced contralateral spread may represent a key target of CPZ-induced impairments. Furthermore, the callosal fibers in the anterior region of the brain follow a curved or angled trajectory relative to the coronal slice plane. This slice-specific effect may reflect anatomical or functional differences within the CC, rendering some areas more susceptible to CPZ toxicity (Steelman et al., 2012). For instance, unique anatomical features or specific connectivity patterns could underlie the heightened vulnerability of SL3 compared with other positions in the present set of slice preparations.

### Discrepancies between functional and structural recovery

Histological evaluation, including FMG and anti-PLP antibody staining, revealed a significant reduction in myelination following CPZ treatment, with only partial recovery in the remyelination phase (Matsushima and Morell, 2001). In contrast, the VSDI showed almost complete functional recovery of interhemispheric propagation in SL3 after CPZ cessation. This discrepancy between structural and functional restoration suggests the involvement of compensatory mechanisms that enable functional connectivity even in the absence of complete myelin restoration. These mechanisms may involve synaptic plasticity, axonal sprouting, or recruitment of alternative neural pathways that compensate for myelin loss and maintain interhemispheric communication (Phokeo et al., 2002; Kwiecien, 2010).

Additionally, the histological markers of myelination used in this study may not have fully captured aspects of myelin that are functionally relevant for signal propagation. Although myelin integrity and distribution appeared visibly impaired, they may still be sufficient to support functional connectivity through adaptation within local circuits. These findings underscore the necessity of considering functional measures alongside structural assessments because relying solely on histological data may underestimate the true extent of neural recovery and plasticity of the CNS.

### Role of long-range fibers and local microcircuits

The propagation of the neural activity observed in this study may involve both direct interhemispheric connections through the CC and local microcircuit activation within the PFC. While the loss of functional interhemispheric connections has not been extensively documented, several electrophysiological studies have reported increased latency in these connections following demyelination (Bando et al., 2008; Crawford et al., 2009). Notably, in our study, the latency of interhemispheric propagation did not exhibit significant changes, even in the partially affected slices (SL2 and SL4); in SL3, the latency could not be measured because of total loss. The latency differences observed in electrophysiological measurements may not be directly comparable with those measured with VSDI, where the recorded responses primarily reflect excitatory postsynaptic potentials (EPSPs) resulting from mono- and/or multisynaptic connections (Tominaga et al., 2009). These differences could contribute to the nonuniform vulnerability observed across different positions in the PFC, potentially reflecting variations in local connectivity, myelination density, or cellular composition. The significant impairment in interhemispheric connectivity observed in SL3 may be linked to the loss of long-range fibers in the CC; however, recovery of functional communication suggests that local circuits may play a crucial compensatory role. The significant decrease observed at the most ventral edge of cg2 in SL3 may reflect alterations in local microcircuits, which may contribute to the overall loss of interhemispheric propagation. This implies that the intrahemispheric communication visualized in this study may depend more on microcircuit activation when long-range connections are disrupted.

Notably, despite the disruption in interhemispheric communication, no significant changes in the latency of neural propagation were observed, even in slices with significant impairment. This suggests that the reduction in connectivity may be attributed to a loss of functional fibers rather than a decrease in the conduction velocity of the remaining fibers. The redistribution of voltage-gated channels during demyelination and remyelination (Lai and Jan, 2006; Kanda et al., 2019) may also contribute to the maintenance of functional propagation without significantly affecting conduction speed. The contribution of synaptic alterations and changes in GABAergic networks should also be considered, as these factors can modulate connectivity independently of direct conduction properties (Dutta et al., 2006; Werneburg et al., 2020).

### Limitations of the study

This study highlights both the strengths and limitations of using VSDI to assess functional connectivity. One major advantage of VSDI is its ability to provide wide-field, high-resolution functional maps of neural activity, a feat that can be difficult to achieve with genetically encoded voltage indicators without employing transgenic animals (Antic et al., 2016; Platisa and Pieribone, 2018; Knöpfel and Song, 2019). However, bulk staining of the VSD has its limitations, such as preferential uptake into nonexcitable lipid components, particularly in the CC, which prevents direct visualization of neural propagation within the CC owing to the high background fluorescence of these components. In the present study, the inability to obtain physiological data from the CC precluded the assessment of potential impairments in neural conduction within the CC. Additionally, the responses captured by VSDI predominantly reflect EPSPs. This limitation makes it challenging to differentiate between the contribution of monosynaptic and polysynaptic connections in interhemispheric propagation.

### Implications for MS and future directions

The findings of this study provide significant insights into the relationship between demyelination and functional recovery of the CNS. The discrepancy between the structural and functional recovery observed in CPZ-fed mice emphasizes the importance of integrating physiological assessments with structural markers, such as myelination. Functional imaging techniques, such as VSDI, can reveal compensatory mechanisms that structural evaluations may miss, offering a more comprehensive understanding of the CNS’s capacity for resilience and plasticity.

Future studies should expand on these findings by utilizing tools such as optogenetic stimulation and genetically encoded calcium and voltage indicators to further dissect the contributions of specific pathways and cellular components in both intra- and interhemispheric connectivity (Petreanu et al., 2007; Luo et al., 2018; Inagaki et al., 2019; Li et al., 2023; Koga et al., 2024). Moreover, understanding the role of local microcircuits in maintaining functional connectivity in the face of demyelination could provide new therapeutic targets for enhancing recovery in patients with MS. Including female participants and studying sex differences will also be crucial for developing treatments that are effective across sexes.

In conclusion, CPZ-induced demyelination leads to significant disruptions in ACC connectivity, particularly affecting interhemispheric communication. Additionally, functional recovery was observed even in the absence of complete myelin restoration, suggesting the involvement of compensatory mechanisms that maintain neural communication. These findings underscore the importance of incorporating functional measures alongside structural assessments in studies on demyelinating diseases. A deeper understanding of how local microcircuits and compensatory mechanisms contribute to recovery may pave the way for more effective therapeutic interventions for MS and related conditions.

## Synthesis

Reviewing Editor: Mark Sheffield, University of Chicago Division of the Biological Sciences Decisions are customarily a result of the Reviewing Editor and the peer reviewers coming together and discussing their recommendations until a consensus is reached. When revisions are invited, a fact-based synthesis statement explaining their decision and outlining what is needed to prepare a revision will be listed below. The following reviewer(s) agreed to reveal their identity: Srdjan Antic, Thomas Knopfel.

Synthesis of Reviews

The manuscript presents a well-designed and methodologically innovative study using voltage-sensitive dye imaging (VSDI) to explore the effects of cuprizone-induced demyelination on interhemispheric communication in the anterior cingulate cortex (ACC). Both reviewers commend the study for its originality, the high quality of the VSDI data, and its potential to provide valuable insights into functional recovery mechanisms in multiple sclerosis (MS) models.

Reviewer 1 emphasizes the importance of addressing technical and interpretational aspects of the findings to enhance the manuscript's rigor and impact. Suggestions include clarifying limitations of the study, particularly the inability to directly record signals from the corpus callosum (CC) using VSDI, and discussing potential technical artifacts or alternative explanations for slice-specific vulnerabilities (e.g., SL3). Reviewer 2 agrees with the merit of the study and suggests that the focus remain on the robust dataset, with speculative discussions kept to a minimum unless supported by the current findings.

Both reviewers agree that revisions are necessary to refine the manuscript's presentation and interpretation of results. Specific areas of improvement include clearer articulation of the methods, more precise positioning of the findings within the broader literature, and minor revisions to enhance figure clarity and consistency.

Decision: A "Revise - Editor Review Only" outcome is recommended, as the required revisions are straightforward and do not necessitate further re-review. The detailed comments from both reviewers that follow will provide clear guidance for addressing these points and ensuring the manuscript's clarity and impact.

Reviewer 1:

The authors utilize optical imaging of neuronal voltages (voltage imaging) to investigate the physiological effects of cuprizone-induced demyelination in the mouse cerebral cortex. The experiments were conducted ex vivo using acute brain slices stained with the voltage-sensitive dye Di-4-ANEPPS. Neuronal activity was evoked via extracellular electrode stimulation, while wide-field LED illumination and a high-speed camera (1,000 Hz frame rate) recorded the resulting signals. The optical voltage signals achieved in this study are of exceptional quality, enabling simultaneous recording from multiple locations across the entire brain slice surface, encompassing both hemispheres.

In this well-executed study, the authors analyzed three experimental groups: Untreated Controls, a Cuprizone-Injured Group, and a Recovery Group (post-cuprizone treatment). Their findings provide significant insights into the cuprizone model of multiple sclerosis (MS). Specifically, they report that cuprizone treatment results in a marked impairment of electrical signal propagation between hemispheres, most prominently in a specific coronal plane referred to as "SL3." This impairment was observed as a failure of voltage waves to traverse the corpus callosum in the Cuprizone Group, whereas in the Control Group, signals propagated effectively. Notably, the optical data illuminate both long-range inter-hemispheric projections and local microcircuit activity within the ipsilateral hemisphere.

Although previous studies suggested a significant delay in signal propagation in demyelinated areas, this study demonstrates that the latency of neural signal transmission remains largely unaffected despite the loss of connectivity (reduced synaptic input). Even in SL3, where inter-hemispheric connections were severely impaired, ipsilateral signal latency was comparable to that in control mice.

Additionally, the authors assessed myelin content in the same groups where physiological data were collected. Functional connectivity between hemispheres fully recovered after cessation of the cuprizone diet (5 weeks); however, myelin did not regenerate to baseline levels. Remarkably, despite the absence of substantial remyelination (~75% myelin loss in recovered mice compared to controls), functional connectivity was not only restored but exceeded control levels. These findings underscore the limitations of relying solely on myelin quantification to assess MS progression, as robust functional recovery can occur despite incomplete structural repair.

This study highlights the importance of functional imaging techniques, such as voltage-sensitive dye imaging, in uncovering compensatory mechanisms missed by structural analyses. The discrepancy between structural and functional recovery observed in cuprizone-treated mice underscores the CNS's remarkable capacity for adaptation, suggesting mechanisms such as increased membrane excitability, redistribution of voltage-gated channels, reorganization of receptor-channel complexes, or axonal sprouting as contributors to functional resilience.

Major

1. SL3 Vulnerability and SNR

The authors conclude that inter-hemispheric fibers in SL3 are uniquely susceptible to cuprizone-induced damage. This implies a special molecular or structural property of fibers in SL3 compared to adjacent sections (e.g., SL2). However, no evidence is provided to support this distinction. Are the authors aware of molecular differences between SL3 and neighboring sections?

Additionally, SL3 shows the strongest contralateral ACC signals in control animals, suggesting it may be the optimal signal-to-noise ratio (SNR) locus. Could the pronounced cuprizone effects in SL3 simply reflect superior SNR at this location? The authors should address/discuss this possibility explicitly.

2. Trajectory of Inter-Hemispheric Fibers

The authors claim that cuprizone-induced impairments are particularly prominent in SL3. However, inter-hemispheric fibers do not follow straight paths; they curve significantly, especially in anterior regions of the frontal lobe, before crossing the midline at the corpus callosum. During slice preparation, curved axonal fibers are more likely to be severed than straight ones. Could the observed vulnerabilities in SL3 result from technical artifacts due to slicing rather than true physiological differences?

3. Demyelination in the Corpus Callosum (CC)

The statement that cuprizone-induced demyelination is "most prominent in the corpus callosum" is a strong claim. The authors should provide at least two references with systematic measurements of cuprizone's demyelinating effects across brain regions. If such evidence is unavailable, this statement should be revised or removed.

4. Corpus Callosum Voltage Signals

The manuscript title emphasizes neural communication via the corpus callosum, yet no physiological signals were recorded from the CC itself, likely due to the incompatibility of myelinated axons with voltage-sensitive dyes. The authors must acknowledge this limitation explicitly:

- Clearly state that physiological data from the CC could not be obtained.

- Discuss this limitation at the beginning of the Results section, noting the technical challenge caused by myelin-VSD incompatibility.

5. Figure 2B Annotations

In Figure 2B, the large, bold white labels indicating 10 ms increments obscure critical regions, including the corpus callosum. Given the centrality of the CC to the manuscript's focus, these labels should be reduced in size or repositioned to avoid obscuring relevant data. The captions already explain the frame intervals (10 ms), making such labels unnecessary.

6. Mechanisms of Functional Recovery

323 While VSD imaging revealed substantial functional recovery in terms of signal propagation, the underlying myelin structure did not return to control levels, suggesting that the restoration of

function may involve compensatory mechanisms independent of full remyelination.

The reader is left hanging. The discussion on compensatory mechanisms for functional recovery should include a forward reference: "may involve compensatory mechanisms independent of full remyelination (discussed below)." This addition "(discussed below)" would guide readers more effectively.

7. Speculative Future Directions

The authors stated: "Further investigations into the microstructural characteristics, cellular composition, and myelination density of different ACC slices may clarify the mechanisms underlying this nonuniform vulnerability at different positions in the ACC".

These methods that you list may clarify the mechanism, or MAY NOT clarify it.

The suggestion to investigate microstructural and cellular characteristics of different ACC slices is overly speculative. If such methods are pivotal, why were they not employed in this study? Vague recommendations about future research diminish the impact of the discussion. The authors should focus on the current study's findings and comparisons with existing literature, omitting speculative prognoses.

Summary

This manuscript provides compelling evidence for the functional resilience of neural circuits following demyelination, challenging the conventional emphasis on structural restoration. Addressing the major comments above will enhance the manuscript's clarity and rigor, ensuring that its conclusions are fully supported by the data.

Reviewer 2:

The authors study the effect of Cuprizone treatment (and withdraw of treatment) on the propagation of electrical activity via the corpus callosum in a mouse brain slice preparing using voltage-sensitive dye imaging. The authors found disruptions in PFC connectivity within and between hemispheres in CPZ-fed mice. Notably propagation speed was not affected. When studying the brain slices prepared from mice after withdraw of Cuprizone treatment a disconnected between myelin stain and function was noted. To my knowledge these are original findings on a relevant issue.

This is a well-designed, conducted and written-up study. I have no major concerns.

Minor comments

Line 222: For readability, please introduce the term "REC mice" even so "REC group" was introduced in the methods section.

In figure 1, it appears that the response was of shorter duration at the side of stimulation in the slice from the treated mouse (C versus B and D). A comment on this may be useful.

## Author Response

2024-12-02 Professor Mark Scheffield Reviewing Editor eNeuro Dear Professor Scheffield:

We wish to re-submit the manuscript titled "Optical assay of the functional impact of cuprizone-induced demyelination and remyelination on interhemispheric neural communication in the anterior cingulate cortex via the corpus callosum." The manuscript ID is eN-MNT-0511-24.

We thank you and the reviewers for your thoughtful suggestions and insights. The manuscript has benefited from these insightful suggestions. I look forward to working with you and the reviewers to move this manuscript closer to publication in eNeuro.

The manuscript has been rechecked and the necessary changes have been made in accordance with the reviewers' suggestions. The revisions in the manuscript are indicated by yellow highlights, and our point-by-point response to the reviewers' comments are given below. The reviewers' comments are presented in bold italic text, followed by our responses, which are written in blue.

Thank you for your time and consideration.

Sincerely, Takashi Tominaga ------------------------ Reviewer 1:

The authors utilize optical imaging of neuronal voltages (voltage imaging) to investigate the physiological effects of cuprizone-induced demyelination in the mouse cerebral cortex. The experiments were conducted ex vivo using acute brain slices stained with the voltage-sensitive dye Di-4-ANEPPS. Neuronal activity was evoked via extracellular electrode stimulation, while wide-field LED illumination and a high-speed camera (1,000 Hz frame rate) recorded the resulting signals. The optical voltage signals achieved in this study are of exceptional quality, enabling simultaneous recording from multiple locations across the entire brain slice surface, encompassing both hemispheres.

In this well-executed study, the authors analyzed three experimental groups: Untreated Controls, a Cuprizone-Injured Group, and a Recovery Group (post-cuprizone treatment). Their findings provide significant insights into the cuprizone model of multiple sclerosis (MS). Specifically, they report that cuprizone treatment results in a marked impairment of electrical signal propagation between hemispheres, most prominently in a specific coronal plane referred to as "SL3." This impairment was observed as a failure of voltage waves to traverse the corpus callosum in the Cuprizone Group, whereas in the Control Group, signals propagated effectively. Notably, the optical data illuminate both long-range inter-hemispheric projections and local microcircuit activity within the ipsilateral hemisphere.

Although previous studies suggested a significant delay in signal propagation in demyelinated areas, this study demonstrates that the latency of neural signal transmission remains largely unaffected despite the loss of connectivity (reduced synaptic input). Even in SL3, where inter-hemispheric connections were severely impaired, ipsilateral signal latency was comparable to that in control mice.

Additionally, the authors assessed myelin content in the same groups where physiological data were collected. Functional connectivity between hemispheres fully recovered after cessation of the cuprizone diet (5 weeks); however, myelin did not regenerate to baseline levels. Remarkably, despite the absence of substantial remyelination (~75% myelin loss in recovered mice compared to controls), functional connectivity was not only restored but exceeded control levels. These findings underscore the limitations of relying solely on myelin quantification to assess MS progression, as robust functional recovery can occur despite incomplete structural repair.

This study highlights the importance of functional imaging techniques, such as voltage-sensitive dye imaging, in uncovering compensatory mechanisms missed by structural analyses. The discrepancy between structural and functional recovery observed in cuprizone-treated mice underscores the CNS's remarkable capacity for adaptation, suggesting mechanisms such as increased membrane excitability, redistribution of voltage-gated channels, reorganization of receptor-channel complexes, or axonal sprouting as contributors to functional resilience.

Major 1. SL3 Vulnerability and SNR The authors conclude that inter-hemispheric fibers in SL3 are uniquely susceptible to cuprizone-induced damage. This implies a special molecular or structural property of fibers in SL3 compared to adjacent sections (e.g., SL2). However, no evidence is provided to support this distinction. Are the authors aware of molecular differences between SL3 and neighboring sections? Additionally, SL3 shows the strongest contralateral ACC signals in control animals, suggesting it may be the optimal signal-to-noise ratio (SNR) locus. Could the pronounced cuprizone effects in SL3 simply reflect superior SNR at this location? The authors should address/discuss this possibility explicitly.

Response: Thank you for your insightful comments regarding the signal-to-noise ratio (SNR) as a potential factor influencing the pronounced effects of cuprizone observed in SL3. In the revised manuscript, we have explicitly addressed this point by acknowledging that SL3 may exhibit higher SNR in terms of optical signals, which could enhance the visibility of interhemispheric propagation under control conditions. However, we argue that SNR alone is insufficient to explain the heightened vulnerability of SL3 to cuprizone-induced impairments, because near-symmetrical ipsilateral and contralateral responses across slices in control conditions suggest consistent data quality. We have also discussed potential anatomical and physiological factors such as connectivity density and callosal fiber trajectories, which likely contribute to this slice-specific effect. Please also see our response to comment #2.

We have included the relevant explanation in the subsection titled "Rostro-caudal position-dependent vulnerability and non-uniform effects" in the Discussion section.

2. Trajectory of Inter-Hemispheric Fibers The authors claim that cuprizone-induced impairments are particularly prominent in SL3. However, inter-hemispheric fibers do not follow straight paths; they curve significantly, especially in anterior regions of the frontal lobe, before crossing the midline at the corpus callosum. During slice preparation, curved axonal fibers are more likely to be severed than straight ones. Could the observed vulnerabilities in SL3 result from technical artifacts due to slicing rather than true physiological differences? Response: Thank you for highlighting the potential influence of slicing artifacts on the observed vulnerability in SL3, particularly due to the curved trajectory of interhemispheric fibers in the anterior corpus callosum (CC). We acknowledge that slicing artifacts can affect anatomical integrity, especially for fibers with non-linear trajectories, and we agree that this is an important consideration when interpreting slice-specific results.

In our revised discussion in the subsection titled "Rostro-caudal position-dependent vulnerability and non-uniform effects" in the Discussion section, we have explicitly addressed this possibility. While the curved trajectory of CC fibers may indeed increase their susceptibility to mechanical damage during slice preparation, we have included the following statement: "This observed vulnerability does not necessarily indicate molecular or physiological uniqueness in this brain region." This acknowledgment underscores our recognition of potential technical limitations while maintaining the focus on biological implications.

3. Demyelination in the Corpus Callosum (CC) The statement that cuprizone-induced demyelination is "most prominent in the corpus callosum" is a strong claim. The authors should provide at least two references with systematic measurements of cuprizone's demyelinating effects across brain regions. If such evidence is unavailable, this statement should be revised or removed.

Response: Thank you for highlighting the need for caution before claiming that cuprizone-induced demyelination is "most prominent in the corpus callosum" (CC). In response, we have revised the sentence in the Introduction to reflect the current evidence more accurately and included appropriate references to support the statement. The revised sentence is as follows: "Cuprizone's demyelinating effects are prominent across various brain regions, with particular vulnerability observed in the corpus callosum (CC) (Hiremath et al., 1998; Jurevics et al., 2002, 2001; Vega-Riquer et al., 2019), a major structure responsible for communication between both cerebral hemispheres, and this targeted demyelination significantly impairs higher brain functions." We believe this revision addresses your concerns by toning down our claim and providing systematic citations to substantiate the role of the CC as a particularly vulnerable structure in cuprizone-induced demyelination.

4. Corpus Callosum Voltage Signals The manuscript title emphasizes neural communication via the corpus callosum, yet no physiological signals were recorded from the CC itself, likely due to the incompatibility of myelinated axons with voltage-sensitive dyes. The authors must acknowledge this limitation explicitly:

- Clearly state that physiological data from the CC could not be obtained.

- Discuss this limitation at the beginning of the Results section, noting the technical challenge caused by myelin-VSD incompatibility.

Response: Thank you for emphasizing the importance of the limitation regarding the lack of physiological data from the corpus callosum (CC). In response, we have revised the manuscript to ensure this limitation is clearly acknowledged and explained across relevant sections. The revisions are shown below.

1. Abstract and Introduction:

We have explicitly stated that physiological data from the CC could not be obtained because of incompatibility of voltage-sensitive dye (VSD) with myelinated axons. The revised text reads as follows:

Abstract "Although we could not record physiological signals from the corpus callosum, the results demonstrated a notable impairment in inter-hemispheric connections within the ACC via the corpus callosum, with the most pronounced loss observed in a specific coronal slice among a series of slices examined." Introduction "Although VSDI cannot record physiological data from the CC because of technical limitations, the ACC, which plays a central role in coordination of cognitive and affective responses, remains an ideal target for studying cuprizone-induced demyelination using VSDI to capture its functional dynamics." 2. Results:

At the beginning of the Results section, we have included a clear explanation of this technical limitation as follows: "VSD preferentially incorporates into lipid-rich components, leading to elevated background fluorescence in myelinated axons, such as those in the CC. Consequently, we did not record physiological data from the CC because of elevated background fluorescence. Instead, we focused on intra- and interhemispheric neural signal propagation within the ACC." 3. Discussion:

In the subsection titled "Limitations of the study", we have introduced the following sentence: "In the present study, the inability to obtain physiological data from the CC precluded the assessment of potential impairments in neural conduction within the CC." These revisions ensure that the limitation is explicitly acknowledged and integrated into the manuscript. We appreciate your valuable feedback, which has helped us improve the clarity and transparency of our study.

5. Figure 2B Annotations In Figure 2B, the large, bold white labels indicating 10 ms increments obscure critical regions, including the corpus callosum. Given the centrality of the CC to the manuscript's focus, these labels should be reduced in size or repositioned to avoid obscuring relevant data. The captions already explain the frame intervals (10 ms), making such labels unnecessary.

Response: Thank you for pointing this out. We have removed most of the large, bold white labels indicating 10-ms increments to avoid obscuring critical regions, including the corpus callosum. The frame intervals are clearly described in the captions, so the removal of these labels ensures better visibility of the data while maintaining clarity.

6. Mechanisms of Functional Recovery 323 While VSD imaging revealed substantial functional recovery in terms of signal propagation, the underlying myelin structure did not return to control levels, suggesting that the restoration of function may involve compensatory mechanisms independent of full remyelination.

The reader is left hanging. The discussion on compensatory mechanisms for functional recovery should include a forward reference: "may involve compensatory mechanisms independent of full remyelination (discussed below)." This addition "(discussed below)" would guide readers more effectively.

Response: Thank you for highlighting this point. We have revised the sentence as follows: While VSDI revealed substantial functional recovery in terms of signal propagation, the underlying myelin structure did not return to control levels, suggesting that the restoration of function may involve compensatory mechanisms independent of full remyelination, as discussed below.

7. Speculative Future Directions The authors stated: "Further investigations into the microstructural characteristics, cellular composition, and myelination density of different ACC slices may clarify the mechanisms underlying this nonuniform vulnerability at different positions in the ACC".

These methods that you list may clarify the mechanism, or MAY NOT clarify it.

The suggestion to investigate microstructural and cellular characteristics of different ACC slices is overly speculative. If such methods are pivotal, why were they not employed in this study? Vague recommendations about future research diminish the impact of the discussion. The authors should focus on the current study's findings and comparisons with existing literature, omitting speculative prognoses.

Response: Thank you for pointing this out. The sentence has been deleted in order to ensure the discussion remains focused on the findings and their relevance to the existing literature.

Summary This manuscript provides compelling evidence for the functional resilience of neural circuits following demyelination, challenging the conventional emphasis on structural restoration. Addressing the major comments above will enhance the manuscript's clarity and rigor, ensuring that its conclusions are fully supported by the data.

Reviewer 2:

The authors study the effect of Cuprizone treatment (and withdraw of treatment) on the propagation of electrical activity via the corpus callosum in a mouse brain slice preparing using voltage-sensitive dye imaging. The authors found disruptions in PFC connectivity within and between hemispheres in CPZ-fed mice. Notably propagation speed was not affected. When studying the brain slices prepared from mice after withdraw of Cuprizone treatment a disconnected between myelin stain and function was noted. To my knowledge these are original findings on a relevant issue.

This is a well-designed, conducted and written-up study. I have no major concerns.

Minor comments Line 222: For readability, please introduce the term "REC mice" even so "REC group" was introduced in the methods section.

Response: Thank you for your valuable comments and suggestions. To enhance readability, we have revised the Methods section to explicitly define "REC mice" and have also ensured consistency in the terminology for all experimental groups ("control mice," "CPZ mice," and "REC mice"). The revised text reads as follows: "Mice were divided into three groups: a CPZ group, a control group, and a recovery group. Mice in the CPZ group were fed 0.3% CPZ-containing powdered feed for 6 weeks with ad libitum access to food and water (hereafter referred to as CPZ mice). Age-matched mice in the control group were fed powdered feed without CPZ for the same period (hereafter referred to as control mice). To assess the recovery effects after CPZ treatment, the diet for the recovery group was switched back to the standard solid feed for an additional 5 weeks following the 6-week CPZ diet (Lindner et al., 2008; Matsushima and Morell, 2001) (hereafter referred to as REC mice)." In figure 1, it appears that the response was of shorter duration at the side of stimulation in the slice from the treated mouse (C versus B and D). A comment on this may be useful.

Response: Thank you for pointing this out. We have added the following sentence to the Results section to address this point: "The ipsilateral side (left side in the figure where the stimulation was applied) in CPZ mice exhibited a smaller and shorter response than did that in the control and REC mice. This is evident when comparing the red trace to the black (control) and green (REC) traces in Figure 2A, as well as in panel C (CPZ) relative to panels B (control) and D (REC)."
